# Joint application of A-InDels and miniSTRs for forensic personal, full and half sibling identifications, and genetic differentiation analyses in two populations from China

**DOI:** 10.1186/s12864-024-10187-4

**Published:** 2024-04-02

**Authors:** Meiming Cai, Fanzhang Lei, Yanfang Liu, Xi Wang, Hongdan Wang, Weibing Xie, Zi Yang, Shangwu Yang, Bofeng Zhu

**Affiliations:** 1 Guangzhou Key Laboratory of Forensic Multi-Omics for Precision Identification, School of Forensic Medicine, Southern Medical University, Guangzhou, Guangdong China; 2 Laboratory of Fundamental Nursing Research, School of Nursing, Guangdong Medical University, Dongguan, Guangdong China; 3 Key Laboratory of Shaanxi Province for Craniofacial Precision Medicine Research, College of Stomatology, Xi’an Jiaotong University, Xi’an, Shanxi China; 4 School of Forensic Medicine, Southern Medical University, Guangzhou, Guangdong China; 5 School of Business, Macau University of Science and Technology, Macao, China; 6 The people’s hospital of Hezhou, Guangxi, China

**Keywords:** Forensic genetics, InDel, MiniSTR, Hubei Tujia group, Hezhou Han population

## Abstract

**Background:**

Previously, a novel multiplex system of 64 loci was constructed based on capillary electrophoresis platform, including 59 autosomal insertion/deletions (A-InDels), two Y-chromosome InDels, two mini short tandem repeats (miniSTRs), and an Amelogenin gene. The aim of this study is to evaluate the efficiencies of this multiplex system for individual identification, paternity testing and biogeographic ancestry inference in Chinese Hezhou Han (CHH) and Hubei Tujia (CTH) groups, providing valuable insights for forensic anthropology and population genetics research.

**Results:**

The cumulative values of power of discrimination (CDP) and probability of exclusion (CPE) for the 59 A-InDels and two miniSTRs were 0.99999999999999999999999999754, 0.99999905; and 0.99999999999999999999999999998, 0.99999898 in CTH and CHH groups, respectively. When the likelihood ratio thresholds were set to 1 or 10, more than 95% of the full sibling pairs could be identified from unrelated individual pairs, and the false positive rates were less than 1.2% in both CTH and CHH groups. Biogeographic ancestry inference models based on 35 populations were constructed with three algorithms: random forest, adaptive boosting and extreme gradient boosting, and then 10-fold cross-validation analyses were applied to test these three models with the average accuracies of 86.59%, 84.22% and 87.80%, respectively. In addition, we also investigated the genetic relationships between the two studied groups with 33 reference populations using population statistical methods of *F*_ST_, *D*_A_, phylogenetic tree, PCA, STRUCTURE and TreeMix analyses. The present results showed that compared to other continental populations, the CTH and CHH groups had closer genetic affinities to East Asian populations.

**Conclusions:**

This novel multiplex system has high CDP and CPE in CTH and CHH groups, which can be used as a powerful tool for individual identification and paternity testing. According to various genetic analysis methods, the genetic structures of CTH and CHH groups are relatively similar to the reference East Asian populations.

**Supplementary Information:**

The online version contains supplementary material available at 10.1186/s12864-024-10187-4.

## Background

Insertion/deletion (InDel) polymorphisms combine the genetic characteristics of single nucleotide polymorphisms (SNPs) such as short amplified fragments and low mutation rates, with the technical advantages of short tandem repeats (STRs) which can be detected by fluorescent-labeled STR primers, multiplex polymerase chain reaction (PCR) and capillary electrophoresis (CE) technique. Therefore, InDel is a kind of relatively ideal genetic marker for forensic practices, including individual identification, paternity testing and biogeographic origin inference [1–3]. At present, researchers have established many InDel panels for different research purposes, which have good values for forensic applications [4–9], but some of the detection systems also exist several limitations. For example, the Investigator DIPplex kit containing 30 autosomal InDel loci, could be better applied for individual identification in Europeans and Americans, but the kit showed lower polymorphisms in Chinese different populations [10, 11]; Jin et al [2] constructed a novel InDel panel for forensic individual identification in Chinese populations, but it still did not sufficiently detect highly degraded samples due to the length of amplification fragments of 35 InDels ranging from 104 to 304 bp.

Therefore, in order to achieve higher forensic identification efficacy in East Asian populations, especially Chinese populations, and to be suitable for detecting highly degraded biological samples, a novel multiplex system of 64 loci was previously constructed ourselves. The multiplex panel is a novel six-color fluorescent labeled amplification system containing 59 A-InDels, two Y-chromosome InDels, two miniSTRs, and an Amelogenin gene. Among them, the two miniSTR loci of this system are not only helpful for the analysis of degraded DNA sample, but also have potential for mixture stain identification [12]. In addition, the Amelogenin gene and two Y-chromosomal InDels in this system can accurately identify sex of the sample contributor. Previously, forensic efficacy and population genetic analysis based on this system were conducted on Manchu in Inner Mongolia Autonomous Region, Zhuang in Yunnan Province [13], Han in Hunan Province [12], two Tibetan groups in Qinghai Province and Tibet Autonomous Region [14], Chinese Hui and Mongolian groups in Northwest China [15]. The results indicated that the multiplex panel of 64 loci provided the powerful tool for forensic individual identification and paternity testing in Chinese populations. However, the system is still in its infancy, and more InDel data from Chinese populations need to be tested before it can be widely used.

So far, the genetic variations of Chinese Tujia group (CTH) in Enshi Tujia and Miao Autonomous Prefecture, Hubei Province and the Hezhou Han in Guangxi Zhuang Autonomous Region (CHH), China, have not yet been systematically studied. Based on the *CHINA STATISTICAL YEARBOOK 2021* (https://www.stats.gov.cn/sj/ndsj/2021/indexch.htm), the number of Tujia people within China was about 9.59 million, which was the eighth largest nationality in China. The CHH group was 1.65 million, accounting for 82.17% of the resident population in Hezhou city. It is located in the northeast of Guangxi Zhuang Autonomous Region, at the junction of Hunan, Guangdong and Guangxi Provinces. This unique biogeographic location may provide an important scenario for studying genetic interactions among different ethnic groups in the region, with great potential for population genetics-related research.

The language of the Tujia is one of the Tibeto-Burman languages of the Sino-Tibetan language family, which is close to the Yi language and is usually considered an relatively isolated language within the Sino-Tibetan language family [16]. The origin of the Tujia group was differently described, one saying that it was descended from the Ba people who settled in Hunan, Hubei and Guizhou Provinces after the Qin Dynasty; the other saying that it was the integration of the Ba people and the Han people. During the Qing dynasty, the Bureaucratization of Native Officers increased the frequency of interaction between Tujia and Han Chinese [17]. In previous studies, 30 InDels [18] and 21 STRs [19] were used to investigate the genetic relationships between the Tujia group and other populations in China. However, the genetic information currently available for Tujia and Han groups is limited, and this subject still needs to be fully explored. Therefore, exploring the genetic relationships between the CTH, CHH groups and other populations is of great necessity, which will help us to further understand the genetic backgrounds of the Han and Tujia groups. It will also help to complete the genomic database of different molecular genetic markers in Chinese populations.

In this study, a novel system of 64 loci self-developed by our lab was used to evaluate the forensic application efficacies in CTH and CHH groups, including paternity testing, individual discrimination, full and half sibling identifications. The genetic relationships among the two studied groups and other 33 reference populations were also investigated on this basis. We hope that this novel system will help address the forensic challenges faced by individual identification, paternity testing, biogeographic ancestry inference for degradation biological samples in CHH and CTH groups.

## Results

### Genetic polymorphisms of 59 autosomal InDels and two miniSTRs in CTH and CHH groups

The details of the 64 loci in this novel system were illustrated in Supplementary Table 1. The Hardy-Weinberg equilibrium (HWE) tests and linkage disequilibrium (LD) analyses for the 59 A-InDels and two miniSTRs (61 loci in total) in CTH and CHH groups were shown in Supplementary Tables 2–4, respectively. There were 7 loci (rs10540867, rs11283102, rs3030496, rs3083268, rs3988323, rs6145346, and rs67787836) in CTH group, two loci (rs10536238, rs3083268) in CHH group deviated from HWE (*P* < 0.05). But after Bonferroni correction, all loci conformed to HWE, and no significant LDs were found among the pairwise 61 loci. The correlation coefficient (*r*^2^) was also calculated to assess the extent of pairwise LDs among the 59 A-InDels. The *r*^2^ values of pairwise 59 A-InDels for CTH and CHH groups were presented in Supplementary Tables 5–6, respectively. The results showed that the *r*^2^ values for all paired loci in both groups were less than 0.2, indicating that these pairwise loci tended towards linkage equilibrium [20].

**Fig. 1:**
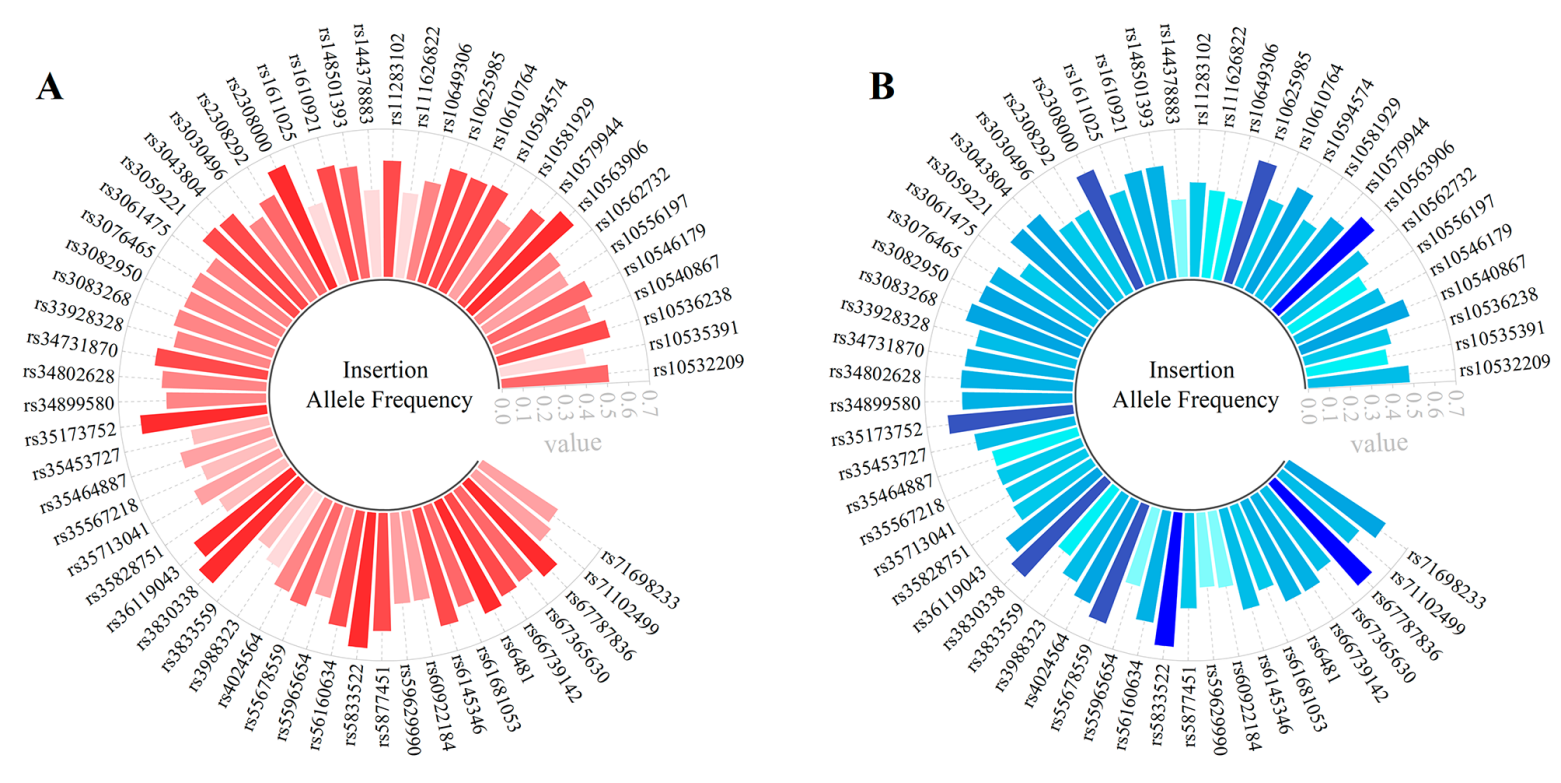
Circular bar graphs of insertion allele frequencies for the 59 A-InDels in CTH group (**A**) and CHH group (**B**). Light red (**A**) and blue (**B**) colors represented smaller insertion allele frequencies, while dark red (**A**) and blue (**B**) colors represented larger insertion allele frequencies

The allele frequencies of the 61 loci in CTH and CHH groups were shown in Supplementary Tables 7–8, respectively. And the insertion allele frequencies of the 59 A-InDels were visualized as the bar chart (Fig. 1). The insertion allele frequencies of the 59 A-InDels ranged from 0.3486 (rs3833559) to 0.6468 (rs3830338, rs10563906) in CTH group (Fig. 1A), and from 0.3529 (rs59629990) to 0.6434 (rs67787836) in CHH group (Fig. 1B). The two miniSTR loci (D1S1656 and D3S1358) in CTH group displayed 11 alleles, with the corresponding allele frequency distributions of 0.0046–0.3028, and 0.0046–0.3440, respectively. These two miniSTR loci also had 11 alleles in CHH group, with their allele frequencies of 0.0074–0.2574, and 0.0515–0.3382, respectively.

**Fig. 2:**
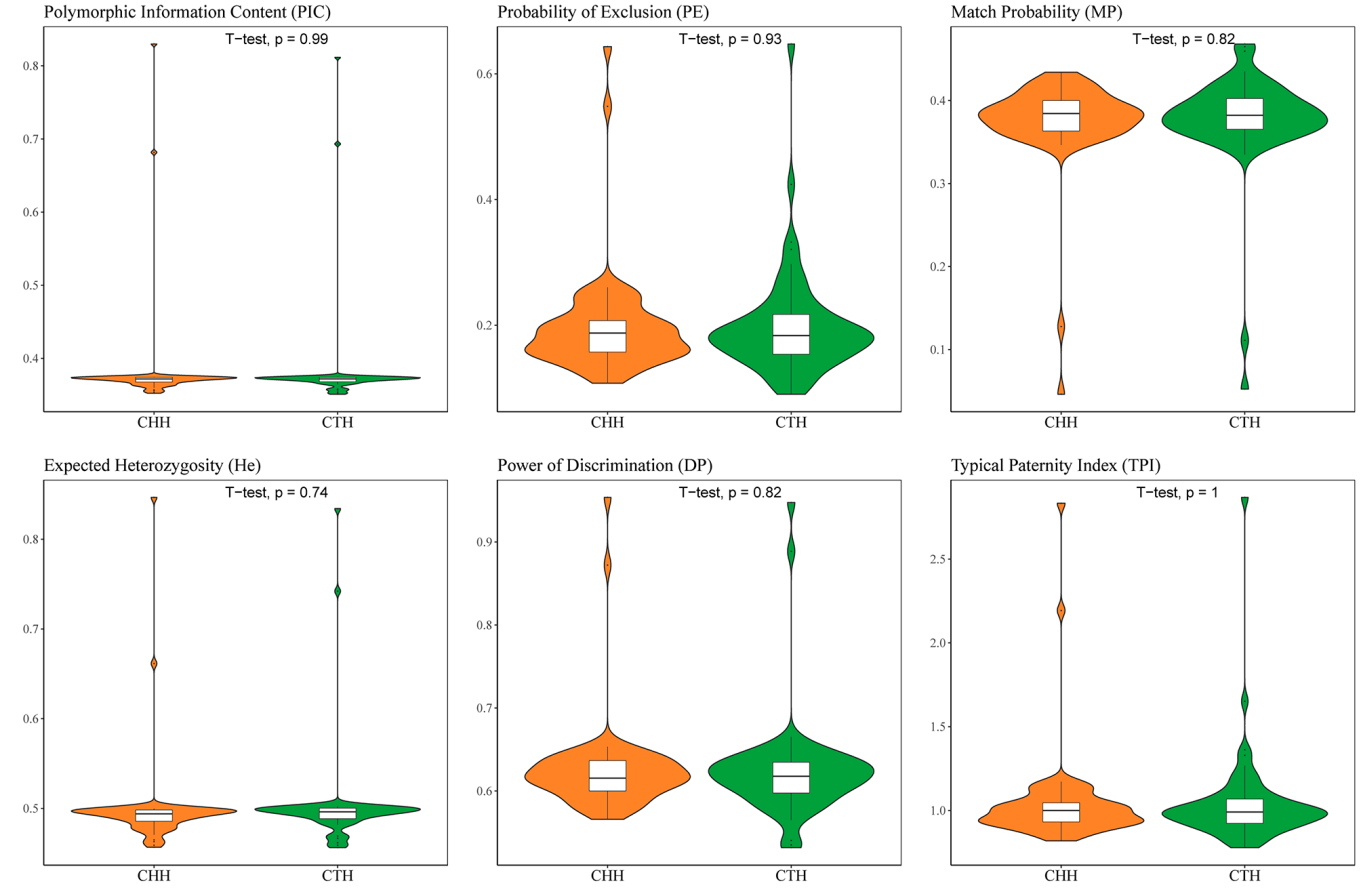
Violin plots of forensic parameters (PIC, PE, MP, He, DP and TPI) of the 61 loci in CTH and CHH groups

The forensic parameters including polymorphism information content (PIC), power of discrimination (DP), match probability (MP), probability of exclusion (PE), observed heterozygosity (Ho), expected heterozygosity (He) and the typical paternity index (TPI) of 61 loci in CTH and CHH groups were shown in Supplementary Table 2 and Fig. 2, respectively. The mean values of PIC, TPI, Ho, DP, PE, MP, He of the 59 A-InDels were 0.3694, 0.9991, 0.4926, 0.6127, 0.1861, 0.3873 and 0.4914 in CTH group, and 0.3695, 0.9902, 0.4910, 0.6155, 0.1828, 0.3845 and 0.4892 in CHH group, respectively. For the 59 A-InDel loci, the largest values of TPI, MP, Ho and PE were at the rs3083268 locus, and rs10540867 locus had the largest DP values in CTH group. For CHH group, rs11283102 and rs33928328 showed the highest values of TPI, PE, and Ho. In the two miniSTR loci (D1S1656 and D3S1358), the highest values of PIC, TPI, DP, PE, Ho and He were found at the D1S1656 locus, with the 0.8116, 2.8684, 0.9476, 0.6476, 0.8257 and 0.8341 in CTH group; 0.8299, 2.8333, 0.9538, 0.6434, 0.8235 and 0.8467 in CHH group.

### Forensic efficiencies of individual identification for 61 loci in CTH and CHH groups

The cumulative MP (CMP), DP (CDP) and PE (CPE) values of the 59 A-InDel loci were 4.2254E-25, 0.99999999999999999999999999999957746, and 0.99999531 in CTH group; and 2.9270E-25, 0.999999999999999999999999707299, and 0.99999369 in CHH group, respectively. With the addition of two miniSTRs, the CMP, CDP and CPE values were 2.4635E-27, 0.99999999999999999999999999754, and 0.99999905 in CTH group; 1.7273E-27, 0.99999999999999999999999999998, and 0.99999898 in CHH group, respectively. As shown in Table 1, the CDPs of the 61 loci were > 0.9999999999999999999999999 and the CPEs were > 0.99999 in 9 East Asian populations.

**Table 1:** Comparisons of the forensic system performances of 61 loci in 9 East Asian populations

Population	Sample size	Cumulative power of discrimination	Cumulative power of exclusion
CTH	109	0.99999999999999999999999999754	0.9999991
CHH	136	0.99999999999999999999999999998	0.9999990
CMI	187	0.99999999999999999999999999758	0.9999987
CZY	99	0.99999999999999999999999999691	0.9999988
CNH	204	0.99999999999999999999999999902	0.9999978
CTT	154	0.99999999999999999999999998494	0.9999990
CTQ	155	0.99999999999999999999999999808	0.9999973
CHN	249	0.99999999999999999999999999990	0.9999982
CMN	222	0.99999999999999999999999999974	0.9999978

### Forensic efficiencies of paternity testing for 61 loci in CTH and CHH groups

**Fig. 3:**
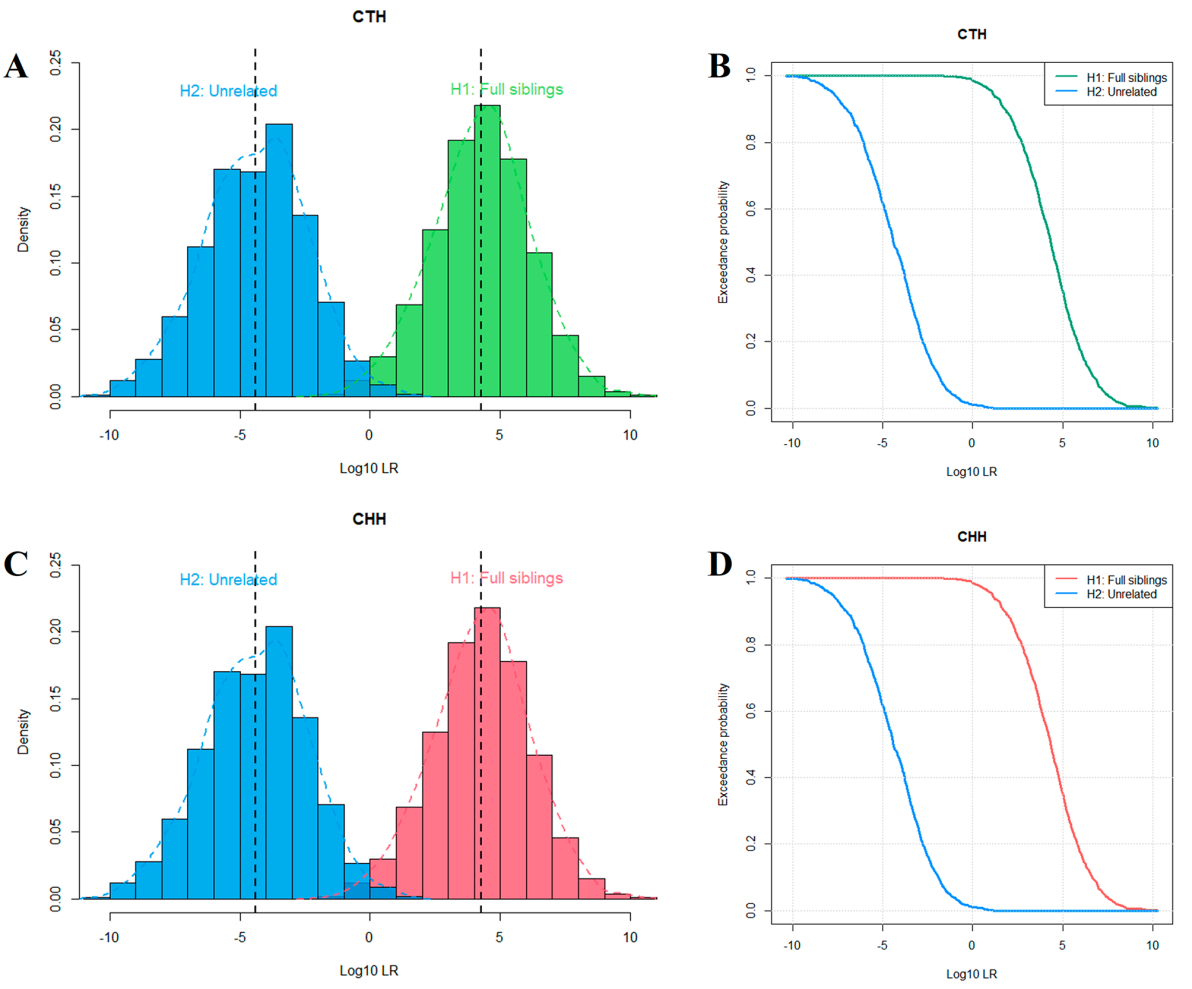
Log_10_(LR) distribution density curves and probability curves of full sibling tests based on allele frequencies of 61 loci. **A, B** Log_10_(LR) distribution and probability curve to distinguish full siblings from unrelated individuals based on allele frequencies of 61 loci in CTH group. **C, D** Log_10_(LR) distribution and probability curve to distinguish full siblings from unrelated individuals based on allele frequencies of 61 loci in CHH group

Simulating 1000 pairs of full sibling identification cases, there are partial overlaps in the Log10(LR) distributions of full siblings and unrelated individuals using the allele frequencies of 61 loci in CTH (Fig. 3A and B) and CHH groups (Fig. 3C and D), respectively. As shown in Supplementary Table 9, when the LR limit was set to 1, the proportions of full siblings and unrelated individuals which could be determined using 61 loci in CTH and CHH groups were 98.60% and 98.40%, respectively, while the false positive rates were 1.10% and 0.60%, respectively. When the LR limits were set to 10, 100, 1000, and 10,000, the accuracy rates were 95.60%, 88.70%, 76.20%, and 57.00% for CTH group; and 95.20%, 89.40%, 78.20%, and 58.10% for CHH group, respectively. In the 1000 pairs of half sibling kinship simulation cases, the Log10(LR) distributions were found to overlap significantly in CTH (Supplementary Fig. 1A and B), and CHH groups (Supplementary Fig. 1C and D), respectively. When LR threshold for half siblings and unrelated individuals was set at 1, the proportions of half siblings which could be distinguished from unrelated individuals in CTH and CHH groups were 70.10% and 71.70%, respectively. In order to more fully validate the efficacy of the multiplex system in identifying full or half siblings, three full and three half sibling real cases were added for further validation, and the genotyping data were displayed in Supplementary Table 10. Based on allele frequencies at 61 loci in the CTH and CHH groups, the Log_10_(LR) values for three true full sibling families were 5.69, 3.70, 8.26; and 5.37, 3.73, 8.39, whereas Log_10_(LR) values for three true half sibling families were 0.99, 2.20, 1.05; and 0.84, 2.18, 0.57, respectively.

### Analyses of population genetic variations between the two studied groups and reference populations

**Fig. 4:**
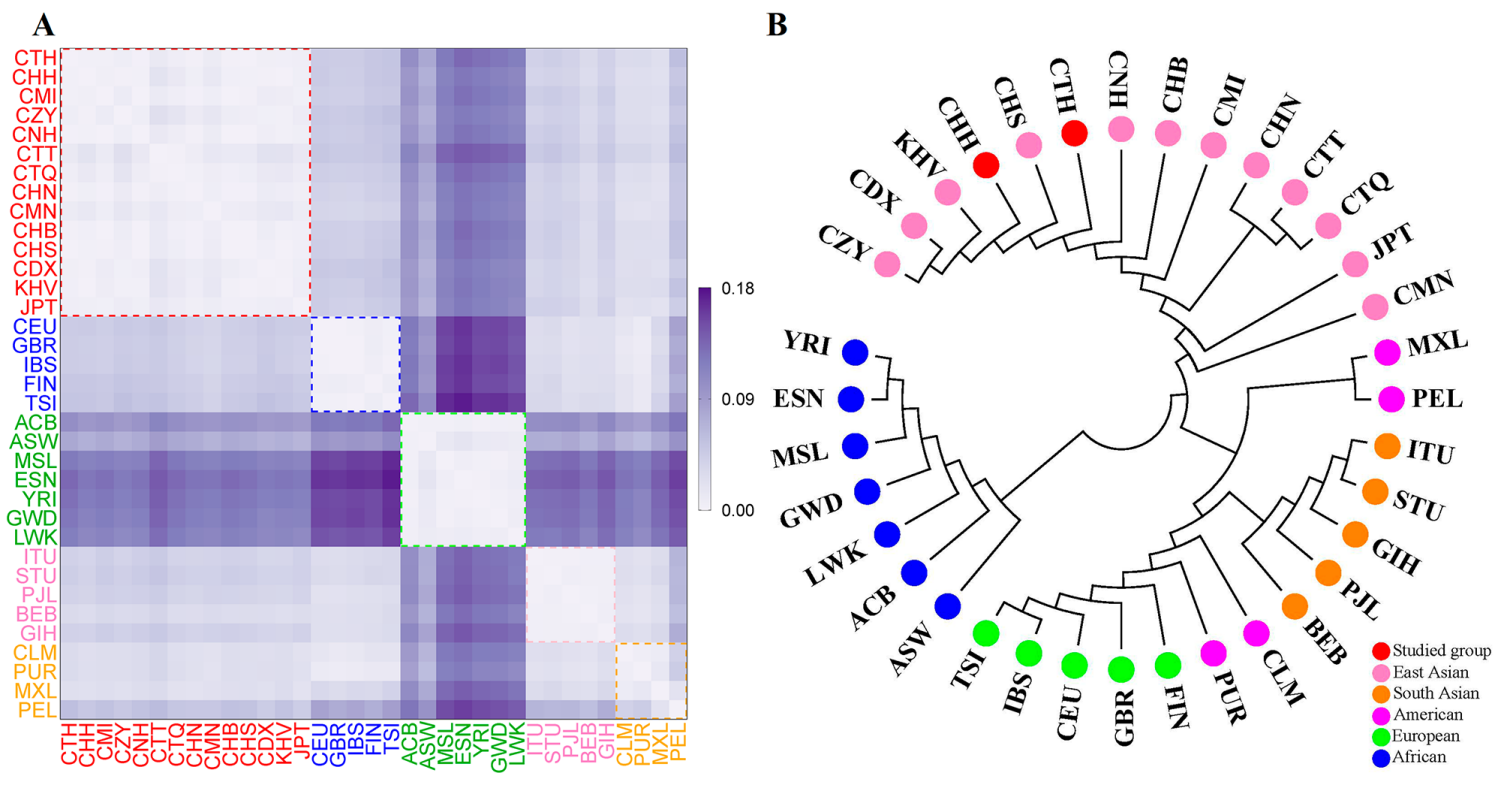
Genetic homogeneity and heterogeneity between two studied groups and 33 reference populations. **A** Heat map of the pairwise *F*_ST_ values based on the 59 A-InDels among the 35 populations. The colors of two studied groups and African populations were close to purple, indicating larger *F*_ST_ values among them. In contrast, the *F*_ST_ values were smaller among East Asian populations and the colors were close to white. **B** The neighbor-joining tree of the 35 populations based on pairwise *F*_ST_ values based on the 59 A-InDel loci

According to Supplementary Table 11 and Fig. 4A, the genetic differences between the two studied groups and the East Asian populations were relatively small. The CTH group exhibited the least genetic difference with the CHS group (*F*_ST_= 0.0000), followed by CNH group (*F*_ST_= 0.0009), and CHN group (*F*_ST_= 0.0013). The CHH group displayed the least genetic difference with the KHV group (*F*_ST_= 0.0000), followed by CZY group (*F*_ST_= 0.0017), and CDX group (*F*_ST_= 0.0018). According to Bonferroni corrected *P* value (*P* > 0.000084), the above paired groups showed no significant differences in genetic distance. While the two studied groups displayed greater genetic differences from the African populations, the greatest genetic differences were found with the ESN group. The *Nei’s D*_A_ genetic distances were also calculated, resulting in Supplementary Table 12 and Fig. 2A. The CTH group was genetically closer to CNH, CHN, and CHS groups, whereas CHH group was genetically closer to KHV, CNH, and CZY groups. In order to further analyze the genetic relationships of 35 populations, a neighbor-joining tree was constructed based on the pairwise *F*_ST_ values (Fig. 4B). The phylogenetic tree had two main branches, herein African populations clustered into one, and Asian, American, and European populations clustered into the other. In addition, the CTH and CHH groups shared the same outermost branching points with CHS and KHV groups, respectively. In addition, phylogenetic reconstruction based on pairwise *D*_A_ distances were confirmed by the genetic pattern revealed by pairwise *F*_ST_ values (Supplementary Fig. 2B).

**Fig. 5:**
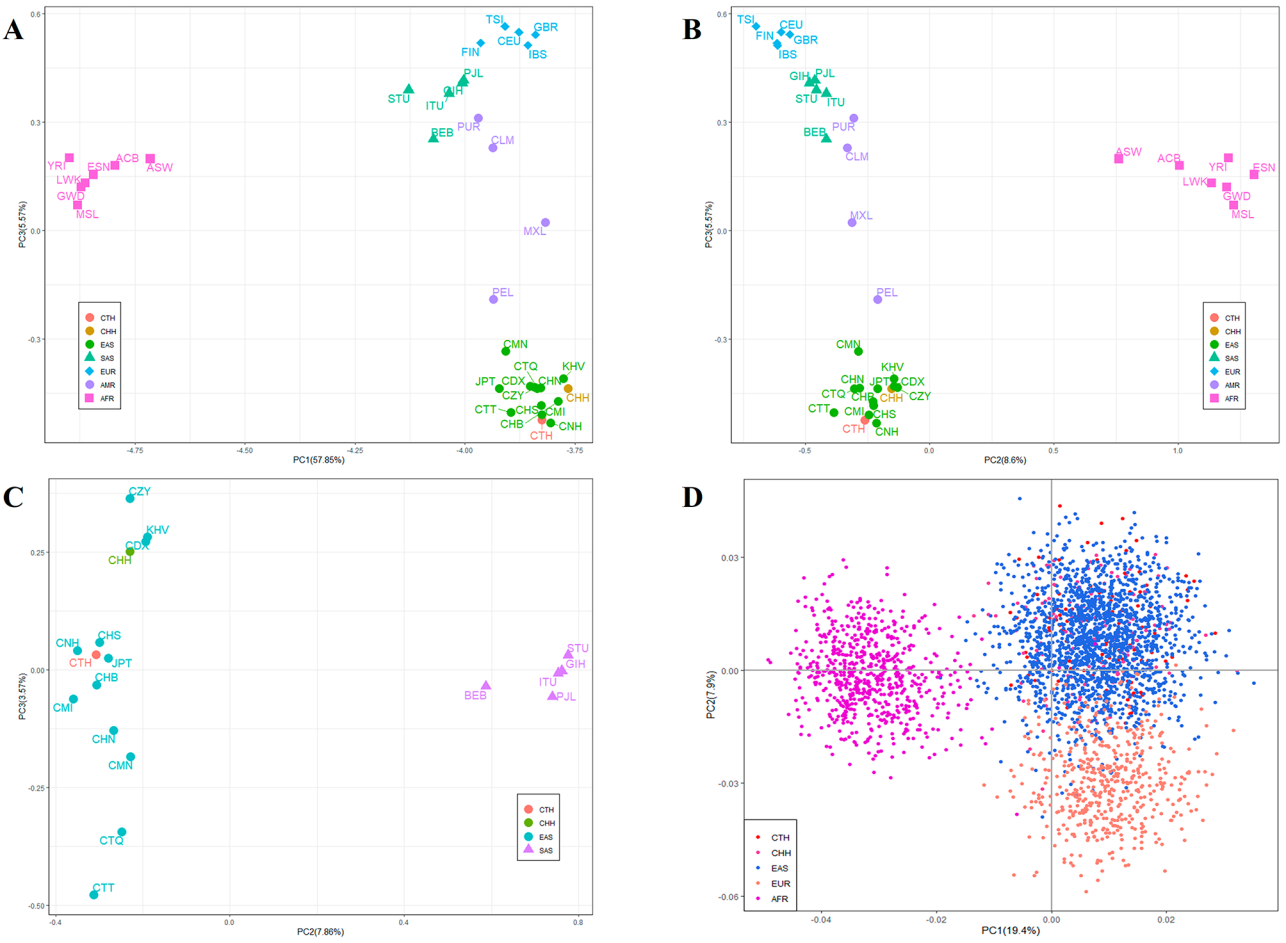
Individual level and population level PCA plots based on the 59 A-InDel loci of the CTH and CHH groups and 33 reference populations. **A** PC1 and PC3 of the five intercontinental populations at population level PCA. **B** PC2 and PC3 of the five intercontinental populations at population level PCA. **C** PC2 and PC3 of the East Asian and South Asian populations at population level PCA. **D** Individual level PCA plot of the CTH, CHH individuals and East Asians, Africans and Europeans at PC1 and PC2

The 72.02% of the total variation could be explained by the three principal components PC1, PC2 and PC3 (57.85%, 8.6%, 5.57%), as illustrated in Fig. 5A and B. Based on PC1 and PC2, it was easy to distinguish between African and non-African populations (American, Asian and European populations), whereas African populations could be differentiated from European, East Asian, and American populations at PC3. Figure 5A and B showed that CTH and CHH groups clustered with 12 East Asian populations due to these populations having similar allele frequency distributions. In addition, it could be found that the East Asian and South Asian populations were clearly separated at PC2 (7.86%) in Fig. 5C. It was also found that the 14 East Asian populations showed some differences in geographical distributions between the south and north in PC3 dimension (3.57%). PCA analysis was performed in this study based on the raw allele genotyping results of 59 A-InDel loci of different individuals from three continents, which could be seen in Fig. 5D. The Fig. 5D showed that all individuals from the same continental region were labeled by the same color, and the three clusters of the East Asian, African and European populations could be identified according to the different colors on the two-dimensional axis, with greater distances between Africa and non-Africa individual clusters. In addition, the individuals of two studied groups clustered with East Asians.

**Fig. 6:**
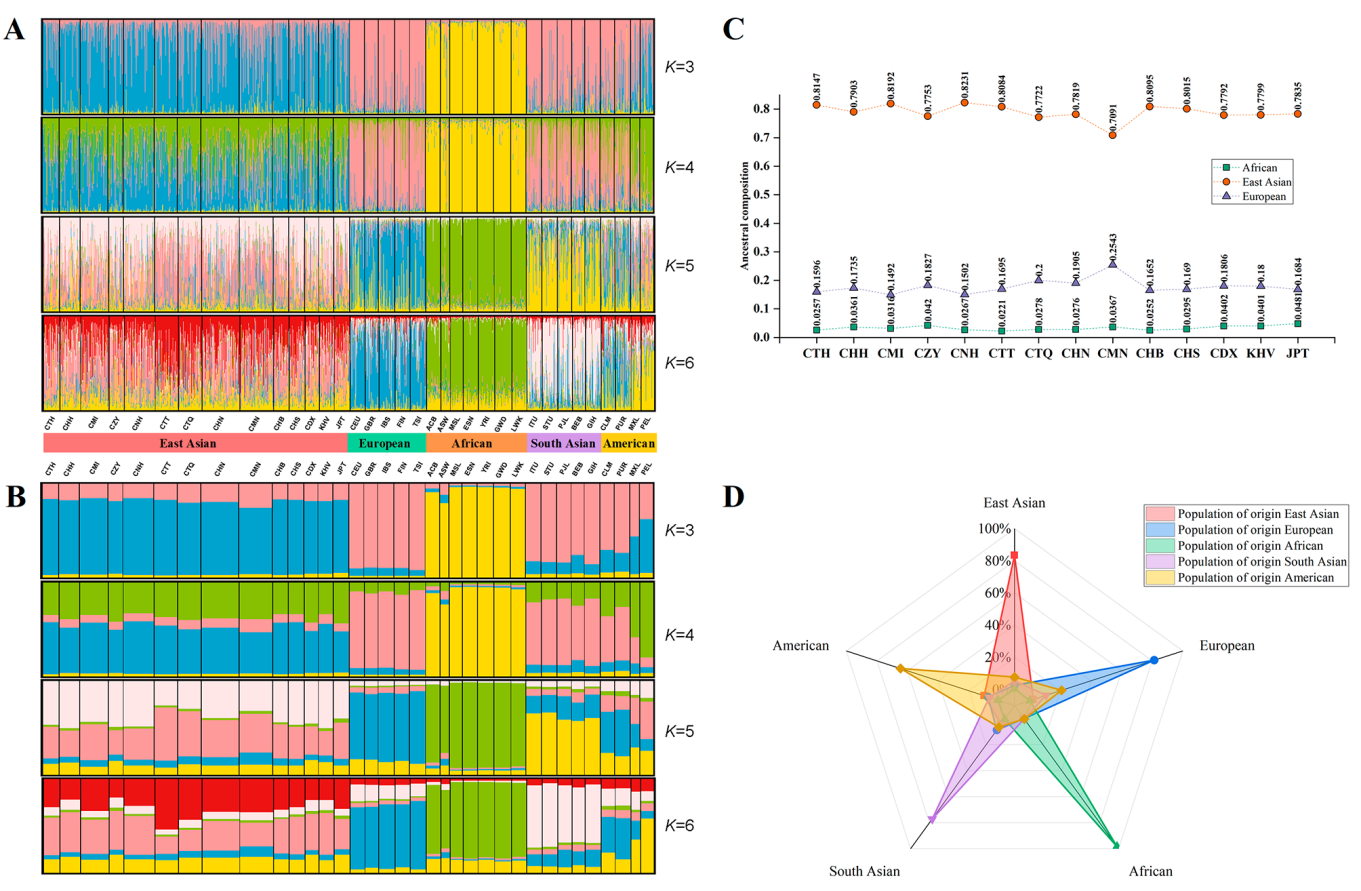
**A, B** Structure analyses of 35 populations (*K* = 3–6) based on the individual level and population level, respectively. **C** Line chart of proportions of ancestral components in East Asian populations when *K* = 3. **D** Radar chart of cross-validation success ratios of the individuals from five continental populations based on the 59 A-InDel loci

In the present research, STRUCTURE analysis was applied to perform ancestral component analyses of 35 populations. The results of genetic STRUCTURE analysis were uploaded to Harvest online tool, and the best *K* value of 3 was obtained (Supplementary Fig. 3). As shown in Fig. 6A and B, the different numbers of colors represented the predetermined values of *K* ancestral components. The proportions of ancestral components for each individual were proportional to the line of the different colored lengths. It could be found that when *K* = 3, East Asians (blue), Africans (yellow) and Europeans (pink) were distinguished from each other, while South Asians and Americans both presented mixtures of Europeans and East Asians ancestral components (pink and blue). And the proportions of ancestral components for 14 East Asian populations were visualized as a line graph (Fig. 6C). It could be observed that the proportions of three ancestral components for CTH and CHH groups were similar. When *K* = 4–6, new colors appeared in East Asians, South Asians and Americans, with different proportions of each ancestral component among them. The accuracy of forensic ancestry inference for different intercontinental populations was assessed on basis of 56 InDel loci by cross-validation. The results of cross-validation success ratios in Fig. 6D showed that 81.31% (blue), 77.10% (purple), 64.55% (yellow), 83.41% (pink) and 98.34% (green) of the biogeographical origins for the individuals from Europe, South Asia, America, East Asia, and Africa were successfully inferred, respectively.

**Fig. 7:**
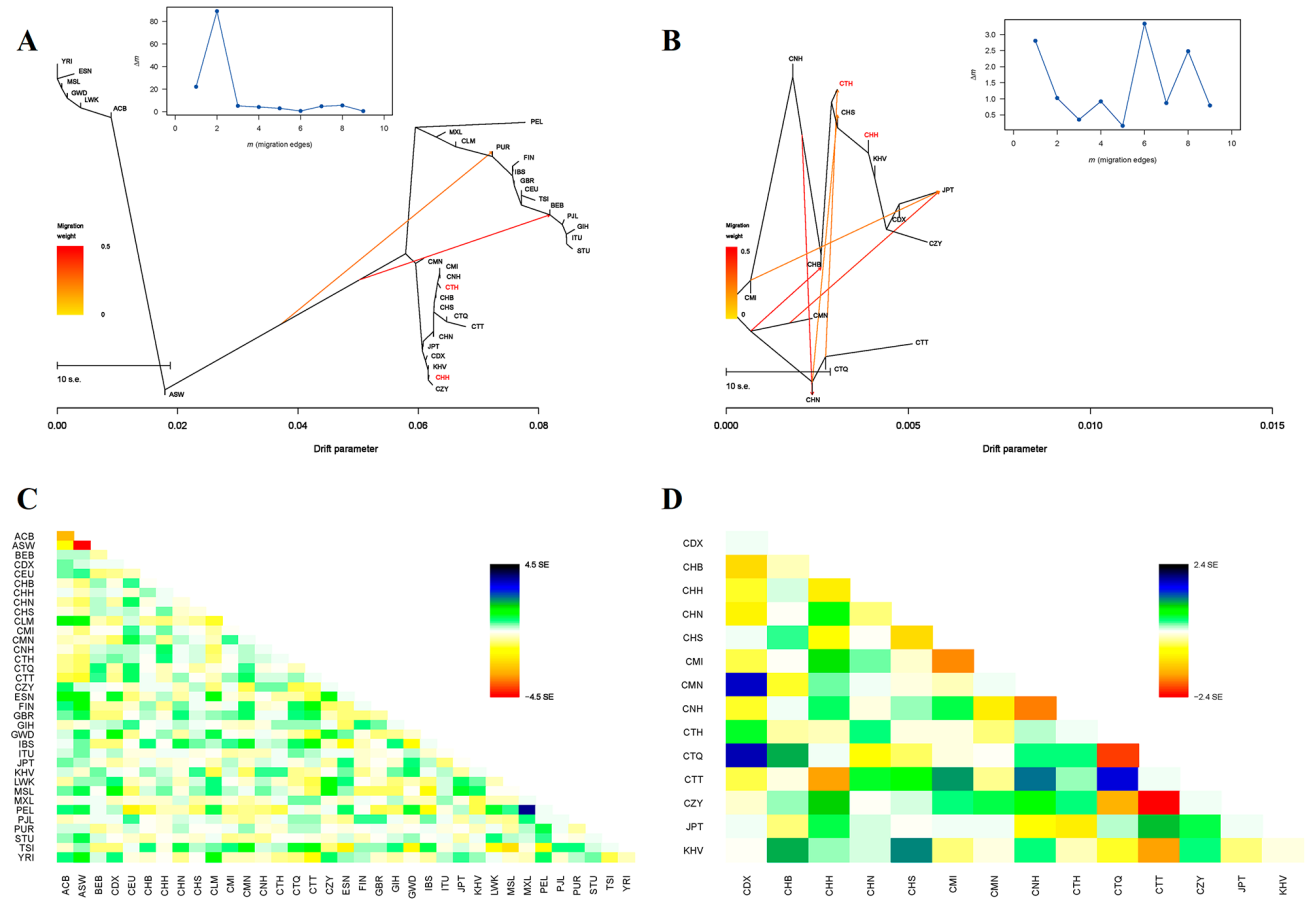
**A, B** Maximum likelihood trees for five intercontinental populations (A) and East Asian populations (B) when the optimal migration events were two (A) and six (B), respectively. The *deltaM* plot was above maximum likelihood tree, with the number of migration events from 1 to 10 (*m* = 1–10). **C, D** Heatmaps of residual fits obtained from the maximum likelihood tree. The color scale was shown on the right. Residuals greater than zero (white) represented that the corresponding populations were closely related and possible admixture events among them

To further explore the phylogenetic relationships among different populations, maximum likelihood trees were constructed using TreeMix software in five intercontinental and East Asian populations, respectively, with the number of migration events ranging from 1 to 10 (*m* = 1–10). The optimal migration event for the 35 populations was two, while the optimal migration event for the East Asian populations was six, as shown in the plots of *deltaM* (Fig. 7A and B). From maximum likelihood tree and residual fit heat map (Fig. 7A and C), we could find that ASW from Africa had gene flow with PUR from America, and BEB from South Asia, respectively. Tibetan, Mongolian groups tended to cluster together, while CTH clustered with CHS, and CHH clustered with KHV (Fig. 7B). The results were in line with the patterns observed in PCA, genetic structure and *F*_ST_ analyses mentioned-above. In addition, gene flow events were observed between CTQ and CTH with high migration weights in Fig. 7B and D. We observed gene flow events from CHN to CHS, and CMN to JPT, which were consistent with the population genetics analysis performed by Fang et al. [15] indicating close correlations between CHN and CHS, and CMN and JPT, respectively.

### Ancestral information inference model constructions and efficacy assessments of 59 A-InDel loci in intercontinental populations

**Fig. 8:**
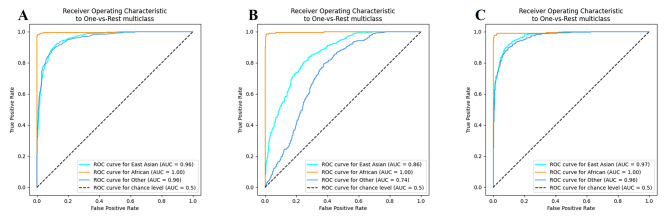
ROC curves of biogeographic ancestry inference results from RF (A), AdaBoost (B), and XGBoost (C) prediction models constructed on the raw data of 59 A-InDel loci from 35 populations. The 35 reference populations were categorized into three groups, East Asian, African, and other continental populations (European, American, and South Asian populations)

In the three-dimensional PCA (Supplementary Fig. 4), it could be seen that the five continent populations were divided into three clusters, i.e., East Asian, African, and other continental population clusters. The cross-validation analyses of five intercontinental populations indicated that the accuracies of biogeographical origin inferences for East Asia and Africa individuals were higher than those of other continental individuals. Therefore, we divided 35 populations into three groups (East Asian, African and other continental populations), and constructed biogeographic ancestry inference models based on random forest (RF), adaptive boosting (Adaboost) and extreme gradient boosting (XGBoost) classifiers. Using One-vs-Rest multiclass strategy, the predicted results were plotted as receiver operating characteristic (ROC) curves, which could be seen in Fig. 8. By observing the ROC plots and comparing the area under curve (AUC) values, it could be found that the three models had the best efficacy in inferring the ancestry of Africans (AUC = 1). Both the RF and XGBoost prediction models had AUC values greater than 0.95 for ancestry inferences for East Asians, Africans, and other continental individuals. The mean accuracies of 10-fold cross-validation tests for the three prediction models were all about 86%, and the XGBoost classifier had the highest accuracy with 87.80%.

## Discussion

Previous studies have confirmed that the six-color fluorescent genotyping system based on capillary electrophoresis platform, which includes 59 autosomal InDels, two Y-chromosome InDels, two miniSTRs and an Amelogenin gene, had good system stability, high sensitivity and specificity, could be used for efficient multiplex amplification detection of forensic degraded biological samples [12, 14]. In this study, we further evaluated and validated the forensic application potential of this novel panel in different ethnic groups in China.

After Bonferroni correction, the 61 loci were conformed to HWE and linkage equilibrium in CTH and CHH groups. Recent studies have shown that the use of Bonferroni correction for HWE tests of multiple loci in forensic population genetics is detrimental to the detection of deviant loci [21]. The results of allele frequency distributions and forensic characteristics in CTH and CHH groups showed that 61 loci exhibited high polymorphisms (PIC > 0.3). And the CDP and CPE of 61 loci in CTH and CHH groups were 0.99999999999999999999999999754 and 0.99999905; 0.99999999999999999999999999998 and 0.99999898, respectively, revealing that 61 loci could be well applied to forensic personal identification and paternity testing in these two groups. Previously, there were many studies on population genetic analyses of Tujia group based on different molecular genetic markers. And the 30 autosomal InDels (Qiagen Investigator DIPplex kit) [18] in Tujia group with CDP and CPE values were 0.9999999999761 and 0.9860, respectively. The 23 STRs (DNA Typer™ 25 kit) [22] with CDP and CPE values were 0.99999999999999999999999753 and 0.99999967 in Tujia group, respectively. Compared with the InDel systems described above, the panel in this study was more suitable for personal identification and paternity testing of Tujia group. Furthermore, we combined 59 A-InDels and two miniSTRs for simulated full sibling and half sibling identifications, and the present results from the distributions of Log_10_(LR) values showed that this system could be used as a valuable tool in full sibling identification cases. In order to validate the simulation results, real cases were also performed, and the LR values for the full sibling identification cases were all greater than 1000, while the half sibling identification cases were all greater than 1. It was demonstrated that the novel system provided valuable conclusions when performing true full sibling identifications. In future studies, we will focus on more InDel loci with high-performance to increase the efficacy in full sibling and half sibling identifications.

The genetic clustering patterns observed by pairwise *F*_ST_ and *D*_A_ values, phylogenetic tree, PCA, and maximum likelihood trees all indicated that CTH and CHH groups had closer genetic relationships with the East Asian populations. For CHH group, the genetic relationships with Southeast Asian populations (CDX, CZY, KHV) were relatively closer. While CTH group maintained close genetic affinities with the surrounding Han Chinese (CNH, CHB, CHS, CHH). He et al. [23] demonstrated that this phenomenon may be due to large-scale population movements and concomitant genetic mixing after the separation of the Tujia group from their common Sino-Tibetan ancestors. The Y-chromosome STR [24], HLA [25] and autosomal InDel [18] have been previously studied to explore the genetic characteristics of the Tujia group, and the present study could further support the previous findings of those researches. Shen et al. [18] explored the genetic polymorphisms of 30 InDels in Tujia group, and found that Tujia group was closely related to Han populations in different regions of China. Yang et al. [24] found that the Tujia and Han populations were genetically closer based on the 17 Y-chromosome STRs. Zhang et al. [25] also found closer genetic relationships between the Tujia and Han populations according to the neighbor-joining phylogenetic tree based on the allele frequencies of HLA-A locus. The ancient Tujia people mainly lived in Hubei, Hunan and Guizhou Provinces, etc., and experienced many wars and migrations [26]. At the same time, since Ming and Qing dynasties, the inflow of neighboring ethnic groups into their settlements led to extensive gene exchanges, objectively promoting the penetration and integration of the various ethnic groups. Both Tujia and Han populations were found to be distributed in the middle of the north-south genetic cline [23], which was also in accordance with their geographical distributions. Interestingly, we found gene flow events between the Tujia and Tibetan groups (CTQ, CTT), both of which belong to the Tibetan-Burmese language of the Sino-Tibetan languages. All of these may be related to their geographical proximity, leading to frequent inter-ethnic gene exchanges: most Tibetans and Tujias reside in the southwestern part of the Chinese territory and in the northwestern parts of Hunan and Hubei Provinces, respectively. However, this panel in this study was not sufficient to confirm the above conclusion, and different types of molecular genetic markers are needed to analyze the genetic relationships between Tujia and Tibetan groups in the future.

In population and individual level PCAs, African, European, and East Asian populations formed separate clusters each other, consistent with their geographic distributions. In contrast, American and South Asian populations were located in the same cluster. STRUCTURE software is one of the most commonly used tools for population genetics analysis, and is often used to test the discrimination efficacy of specific marker combination for studied population. The result of the Harvest online tool showed the optimal *K* value was three, indicating the presence of three main intercontinental populations (East Asians, Africans and Europeans) among all 4019 individuals. The ancestral component distributions of the two studied groups were consistently similar to those of the East Asian populations when *K* = 3–6, indicating the closer relationships between two studied groups and the East Asian populations. The results of the cross-validation analyses for the biogeographical origins of individuals at the five-continent level showed that all the 59 A-InDels were effective in discriminating the ancestry origins of individuals from African, European and East Asian populations, but were less effective when the unknown individuals were from American and South Asian populations. The XGBoost model could achieve the ancestry discriminations for East Asian, African and other continental populations, with average accuracy of 87.80% using 10-fold cross-validation, which was higher than the RF (86.59%) and AdaBoost (84.22%) models. Compared with the RF and AdaBoost models, XGBoost classifier may be more suitable for biogeographic ancestry inference based on InDel genotyping data [27, 28]. A series of population genetic analyses confirmed that the novel system showed good efficacy in inferring ancestry information in African, European and East Asian populations, and reveal that the genetic structures of the two studied groups were more similar to those of East Asian populations.

## Conclusions

The multiplex PCR system with 59 A-InDels, two Y-chromosomal InDels, two miniSTRs and an Amelogenin gene in this study had high efficacy for forensic application in the Hezhou Han population and Hubei Tujia group, which could be used as an effective novel tool for individual identification and paternity testing, and had potential value for full sibling identification case. The relatively similar genetic backgrounds of the Han and Tujia groups were also revealed, while compared to other populations, the Hezhou Han population was closer genetically related to Southeast Asian populations, especially to the Vietnamese Kinh from the 1000 Genomes Project. It was confirmed that the XGBoost classification model constructed based on 59 A-InDel typing data from 35 populations had higher accuracy in inferring ancestral origins of East Asian, African and other continental populations. In summary, this novel system can not only be well used for individual identification and kinship analysis, but also for investigating the genetic relationships and structures among different populations.

## Methods

### Sample preparation

In accordance with the principle of informed consent, peripheral blood samples were collected from 109, and 136 healthy unrelated individuals in CTH, and CHH groups after obtaining their written informed consents, respectively. In addition, blood samples from three full siblings and three half siblings of real cases were collected to validate the efficacy of this multiplex system in identifying full or half siblings. This present study was conducted with strict reference to the principles of human ethical research and approved by the Ethics Committees of Southern Medical University and Xi’an Jiaotong University (No. 2019 − 1039). The 26 continental populations in the 1000 Genomes Project phase III [29] were used as reference populations. In addition, we also added the previously reported groups as reference populations in this research, such as Manchu group in Inner Mongolia Autonomous Region, Zhuang group in Yunnan Province [13], Han population in Hunan Province [12], two Tibetan groups in Qinghai Province and the Tibet Autonomous Region [14], and Hui and Mongolian groups in China [15]. The full names, abbreviations, and sample sizes of the above reference populations could be found in Supplementary Table 13.

### DNA amplification and genotyping

In this study, multiplex amplification assays for the two groups were performed on GeneAmp PCR system 9700 (Applied Biosystems, Foster City, CA, USA) using the self-developed panel of 64 loci, consistent with the amplification program of the previously reported research [15]. Primer design and primer information for all loci were reported by Liu et al. [12]. After PCR amplification, the PCR products of 64 loci were subjected to CE detection on the 3500xL Genetic Analyzer (Applied Biosystems, Foster City, CA, USA). And genotyping results were performed by GeneMapper ID v3.2 (Applied Biosystems, Foster City, CA, USA).

### Statistical analysis

The allele frequencies and forensic parameters of 59 A-InDel loci and two miniSTRs in CTH and CHH groups were calculated using STRAF v1.0.5 online software (http://cmpg.unibe.ch/shiny/STRAF/) [30], and included HWE *p* value, MP, TPI, PE, DP, He, Ho, PIC and LD analysis. The OriginPro v2021 software was used to visualize allele frequencies in the two studied groups, and the ‘ggpubr’, ‘gridExtra’ and ‘ggplot2’ packages in *R* software [31] were used to draw violin plots of forensic parameters. Based on the allele frequencies of 59 A-InDels and two miniSTRs in CTH and CHH groups, 1000 full sibling pairs and 1000 half sibling pairs were simulated using Familias v.3.0 software [32]. LR values for three real full sibling and three half sibling identification cases were also calculated using the Familias v.3.0 software. The LR of the H_0_ hypothesis, that two individuals are full sibling or half sibling pairs, and the H_1_ hypothesis, that two individuals are unrelated, is calculated for validation. Log_10_(LR) distributions of simulated full sibling and half sibling kinship identification cases were plotted using *R* software.

The Arlequin v3.5 software [33] and DISPAN program [34] were used to evaluate the *F*_ST_ and *D*_A_ values of all pairwise populations on basis of 59 A-InDel loci, respectively. The *F*_ST_ and *D*_A_ results of pairwise populations were plotted as the heat maps and neighbor-joining trees using the ‘ggplot2’ package in *R* software and MEGA v7.0 software [35], respectively. Population level PCA plots for 35 populations were constructed based on insertion allele frequencies using the ‘prcomp’ and ‘ggplot2’ packages in *R* software. And individual level PCA analysis was performed on three intercontinental populations (African, European, and East Asian) by PLINK software v1.9 (https://www.cog-genomics.org/plink/1.9/). Three-dimensional PCA at the individual level for 35 populations was visualized using OriginPro v2021 software.

Based on the raw genotyping data of 59 A-InDels, the genetic structures of CTH and CHH groups were inferred by STRUCTURE v2.3.4 software [36]. The parameters “Number of MCMC Repetitions " and “length of burn-in period” were set to 10,000. The number of assumed ancestral clusters was set from 3 to 6, and each *K* value was performed 15 repetitions. The optimal *K* value was obtained using the online web Harvester program (http://taylor0.biolog.ucla.edu/structureHarvester/). The average Q-matrix for each *K* was evaluated by CLUMPP v1.1.2 software [37]. Subsequently, the results of the genetic structure analyses for the 4019 individuals and 35 populations based on the genotyping data of 59 A-InDels were visualized using DISTRUCT v1.1 software [38].

To further explore genetic relationships between the two studied groups and other reference populations, we constructed maximum likelihood trees with predefined mixture event variables using the TreeMix software v1.1 [39]. Cross-validation analyses were conducted on individuals from different continental levels based on the Snipper v3 online tool (http://mathgene.usc.es/snipper/), and the validation results were visualized using OriginPro v2021 software.

We used the SciKit-learn Python package [40] to build machine learning models including RF, Adaboost and XGBoost for biogeographic ancestry inferences based on 59 A-InDel data. And the 35 populations were divided into three groups, i.e., East Asian, African, and other continental populations. These prediction models were built by randomly sampling 70% of the individuals from each group as the training set. The 30% of the remaining individuals were used as the testing set to predict the individual biogeographical origins, and multiclass ROC curves were drawn based on the prediction model results. In addition, all prediction models were tested using 10-fold cross-validation, and the mean value of the 10-fold cross-validation accuracy was applied as the effectiveness assessment index of the prediction model.

### Electronic supplementary material

Below is the link to the electronic supplementary material.

Supplementary Material 1

Supplementary Material 2

## Data Availability

The data which support the findings of this study are available from the corresponding author upon reasonable request. The raw genotyping data of individuals are not publicly available due to ethical restrictions.

## Abbreviations

A-InDel: Autosomal insertion/deletion
SNP: Single nucleotide polymorphism
STR: Short tandem repeat
PCR: Polymerase chain reaction
HWE: Hardy-Weinberg equilibrium
LD: Linkage disequilibrium
PIC: Polymorphism information content
CDP: Cumulative power of discrimination
CMP: Cumulative match probability
CPE: Cumulative probability of exclusion
Ho: Observed heterozygosity
He: Expected Heterozygosity
TPI: Typical paternity index
LR: Likelihood ratio
PCA: Principal component analysis
RF: Random forest
Adaboost: Adaptive boosting
XGBoost: Extreme gradient boosting
ROC: Receiver operating characteristic
AUC: Area under curve
