# Supplementary material for: Joint application of A-InDels and miniSTRs for forensic personal, full and half sibling identifications, and genetic differentiation analyses in two populations from China
Source: BMC Genomics. 2024 Apr 2;25:329. doi: 10.1186/s12864-024-10187-4 (PMC10986087; doi:10.1186/s12864-024-10187-4)
Supplement: Supplementary file 2 — Supplementary Material 2 [file 12864_2024_10187_MOESM2_ESM.docx]

**Supplementary Figures 1-4**


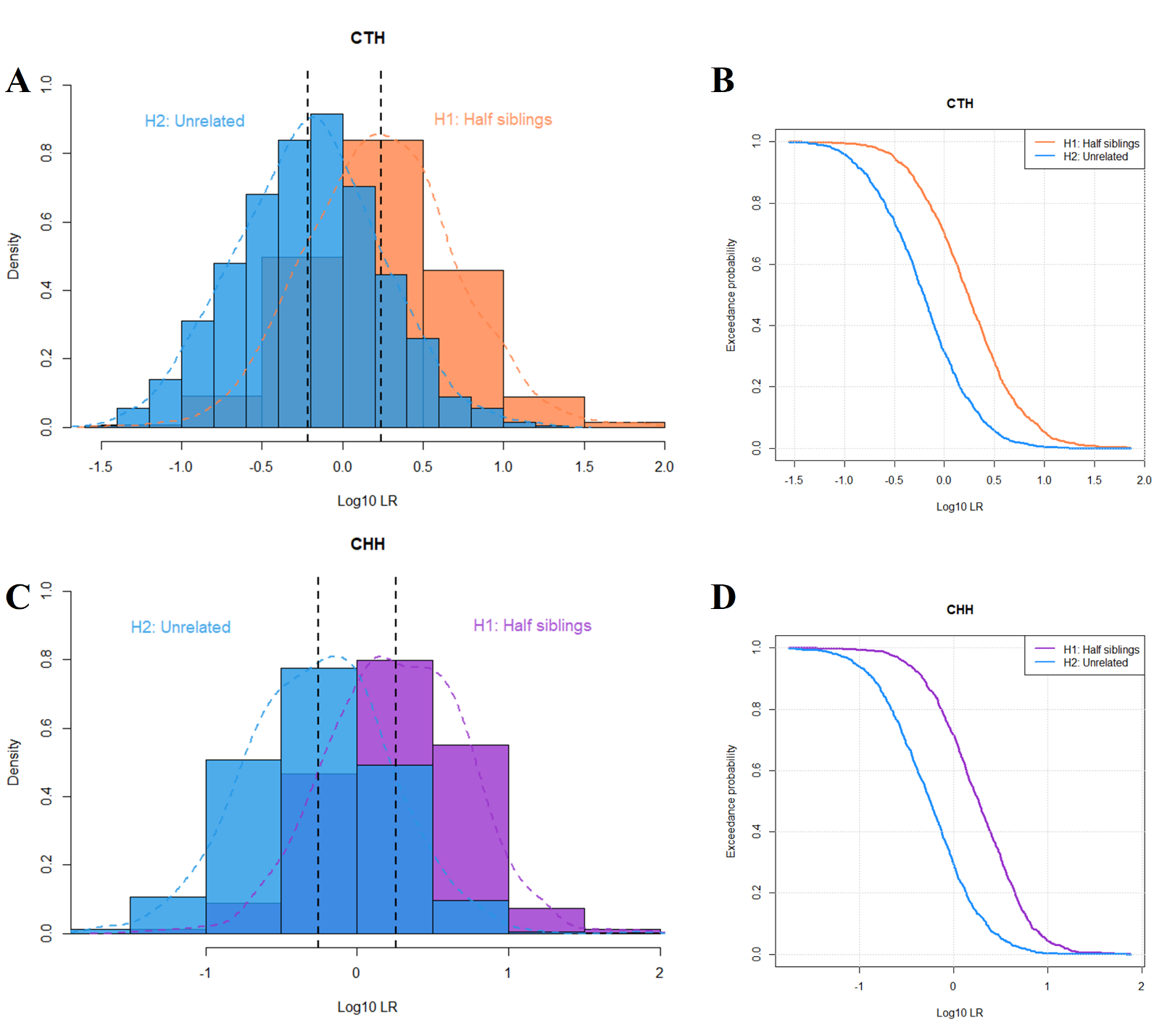


**Supplementary Fig. 1** Histograms of Log_10_LR distributions and probability curves of half sibling tests based on allele frequencies of 61 loci in CTH and CHH groups, respectively. **A, B** Log_10_LR distributions and probability curves to distinguish half siblings from unrelated individuals based on allele frequencies of 61 loci in CTH group. **C, D** Log_10_LR distributions and probability curves to distinguish half siblings from unrelated individuals in CHH group


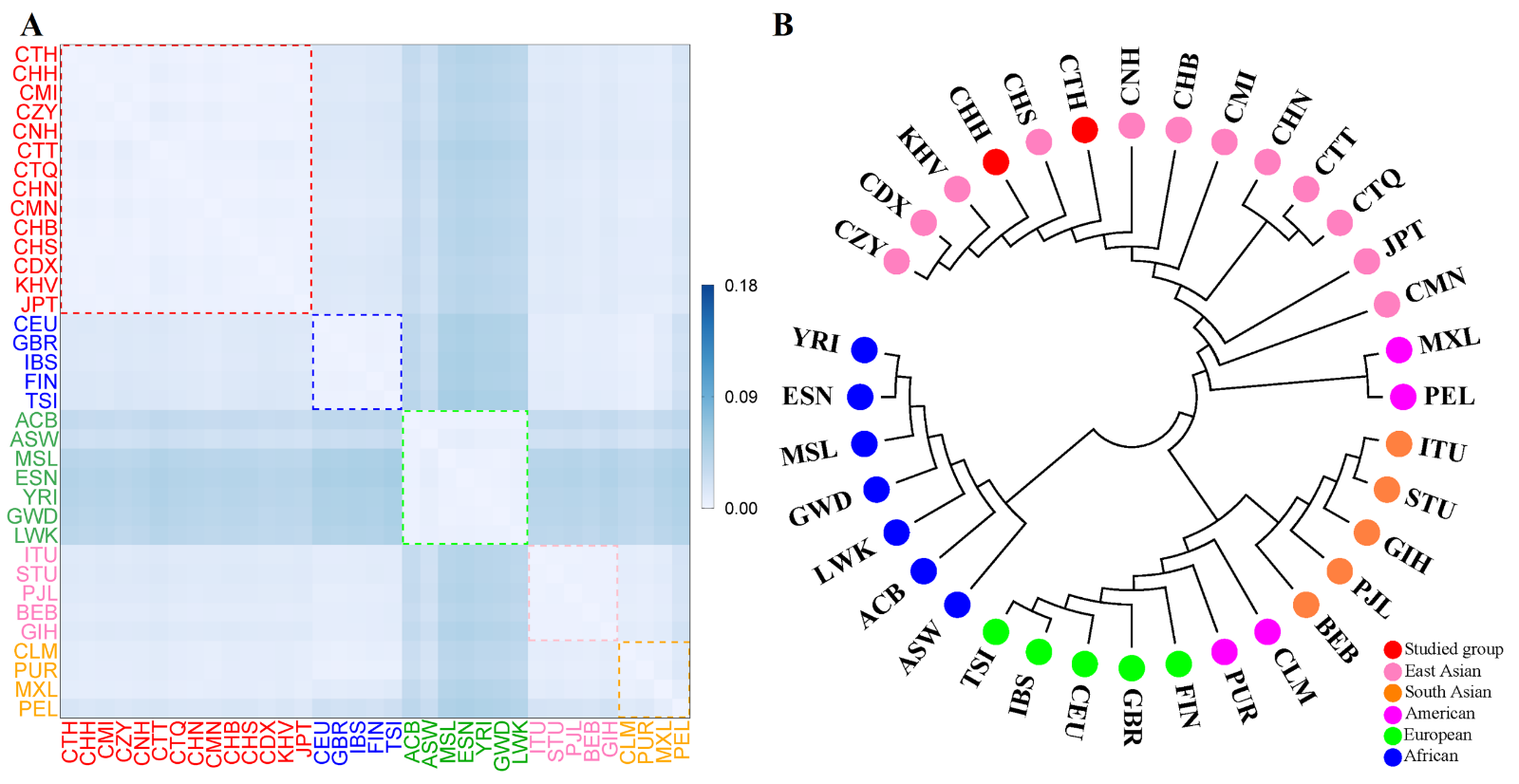


**Supplementary Fig. 2** Genetic homogeneity and heterogeneity among CTH, and CHH groups and 33 reference populations. **A** The heat map of the pairwise *D*_A_ values based on the 59 A-InDels among 35 populations. **B** The neighbor-joining tree using the pairwise *D*_A_ values based on the 59 A-InDel loci among 35 populations


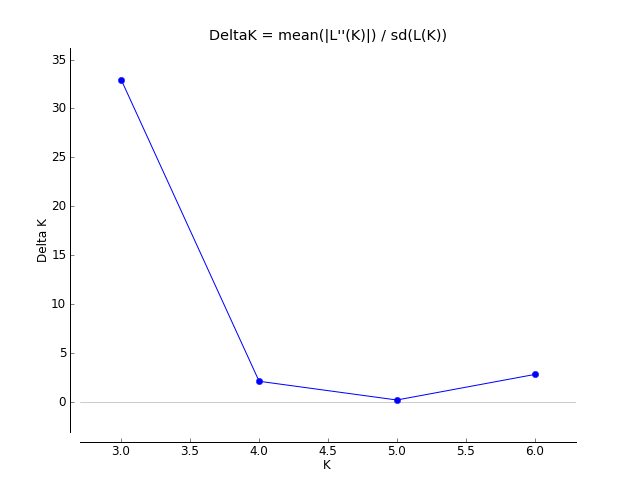


**Supplementary Fig. 3** The optimal *K* value of 3 was obtained by Structure Harvester online software


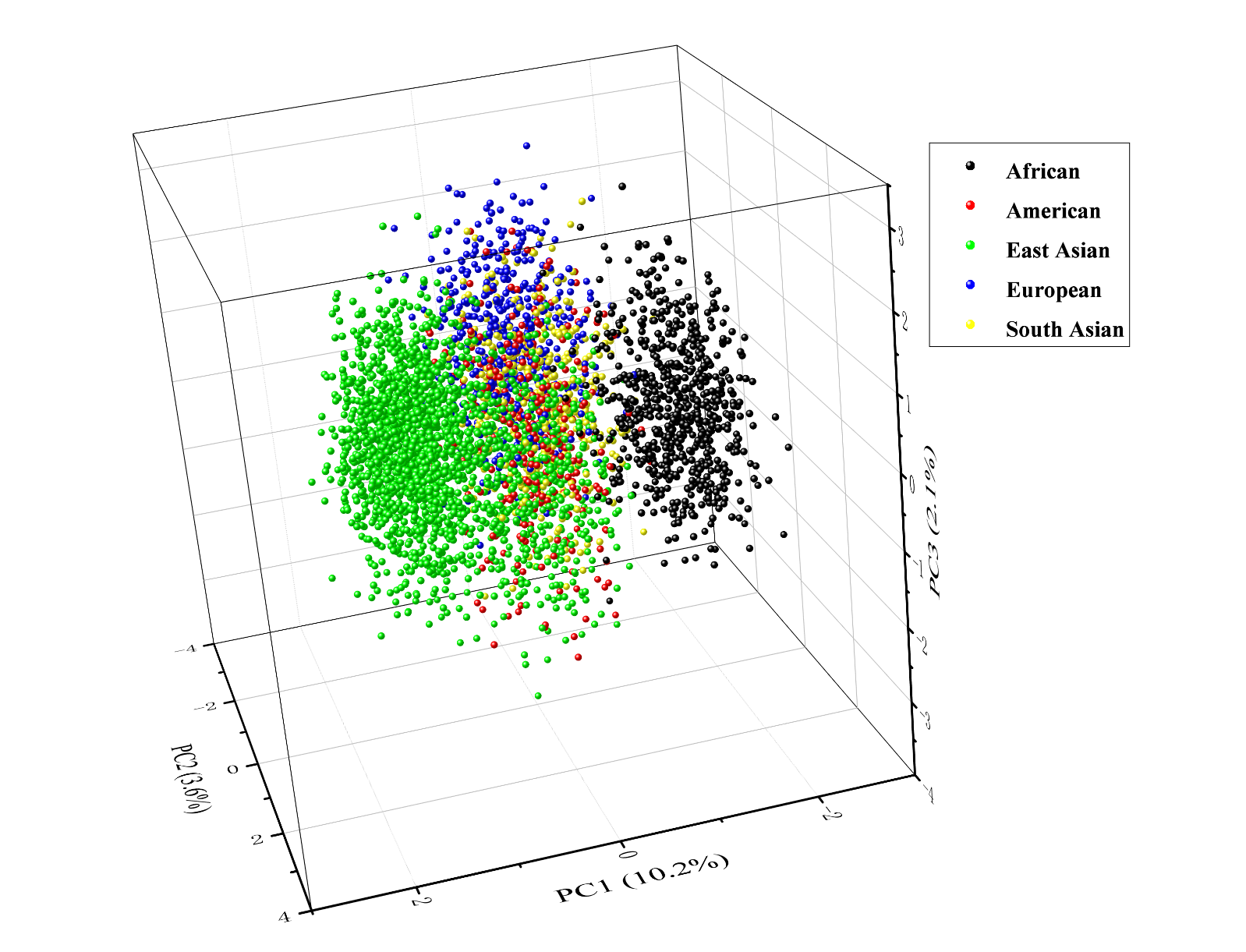


**Supplementary Fig. 4** The three-dimensional PCA on individual-level based on 59 A-InDels genotyping data from the studied CTH, CHH groups and 33 reference populations
